# Explore with Me: Peer Observation Decreases Risk-Taking but Increases Exploration Tendencies across Adolescence

**DOI:** 10.1007/s10964-022-01608-2

**Published:** 2022-05-09

**Authors:** Corinna Lorenz, Jutta Kray

**Affiliations:** 1 Development of Language, Learning and Action, Saarland University, Saarbrücken, Germany; 2 Institute of Psychology, University of Wuppertal, Wuppertal, Germany

**Keywords:** Adolescence, Decision-making, Peer presence, Individual differences, Exploration, Computational modeling

## Abstract

It has been assumed that adolescents increase risk-taking tendencies when peers are present but findings on experimental decision-making have been inconclusive. Most studies focus on risk-taking tendencies, ignoring the effects peer presence can exert over other cognitive processes involved in decision-making, as well as any other underlying developmental and individual differences. In the present study, the trial-by-trial choice behavior was analyzed in a task in which adolescents adjust to dynamically changing risk probabilities. Using Bayesian modeling, the study aimed to infer about peer presence effects on risk-taking tendencies but also on reactions to, exploration of, and learning from positive and negative outcomes of risk-taking. 184 pre- to late adolescents (*M* = 14.09 years, min = 8.59, max = 18.97, *SD* = 2.95, 47% female) conducted the Balloon Analog Risk Task under two conditions: Once alone and once in the presence of a (non-existent) peer observing them virtually. Findings revealed that (a) peer observation reduced risk-taking but increased exploration tendencies and (b) that individual differences modulated this effect. Especially female pre-adolescents increased their openness to explore different choice outcomes when a peer observed their behavior. These results support the assumption that the occurrence and direction of peer influences on risk-taking depend on a person-environment interaction, emphasizing the dynamic role peers play in adolescent risk-taking.

## Introduction

During adolescence, the social environment undergoes dramatic changes. As adolescents increase the time spent with peer, they also increase the engagement in risk behaviors. Even though such observations have commonly been interpreted as adolescents being specifically susceptible to peer influences (Albert et al., 2013), this study set out to directly model the extent to which adolescents are influenced by peer presence during risky decision-making. Most investigations have not employed pure measures of risk-taking tendencies, as many risky decision-making tasks are about about how to deal with uncertainty about risks and possible positive and negative outcomes (Do et al., 2020b). In fact, many risk-taking behaviors, whether in the laboratory or in real-life, are not only about risk-taking tendencies, but how information is utilized to guide decision-making (Silva et al., 2016). Therefore, the purpose of this study is to disentangle which cognitive processes are influenced by the presence of a virtual peer when dynamically changing risk probabilities and benefits can only be experienced.

In the decision-making literature, risk-taking is commonly defined as the tendency to choose options with the greatest variance in outcomes. For example, adolescents are considered risk-prone if they tend to choose options that imply multiple possible outcomes over options with a certain outcome (Figner & Weber, 2011). Furthermore, experimental investigations of risky decision-making allow researchers to directly manipulate the social context of risk-taking behavior. Self-described peer resistance has shown to be low in adolescence and to increase with age (Steinberg & Monahan, 2007). To experimentally quantify and to test developmental differences in peer presence effects, studies introduced either direct peer interaction, passive presence, or observation during risky decision-making. When comparing the effect of peer advice and observation, one study showed increases in risk-taking tendencies in both, adolescents and adults, when a peer encouraged risk-taking in a gambling task. Peer observation, however, only increased adolescents’ risk-taking tendencies, highlighting the importance to distinguish between different peer presence effects (Haddad et al., 2014). Passive peer observation, such as the observation by a friend (Somerville et al., 2019), or unfamiliar and only virtual peer (Haddad et al., 2014, Smith et al., 2014), has been shown to modify adolescent risk-taking tendencies. However, when a peer did not explicitly observe behavior, mere peer presence was sometimes not sufficient to affect risk-taking tendencies in adolescence (Somerville et al., 2019). As such, recent findings about the benefit of peer influence on risk taking have been inconclusive depending on the type of risk and social context of the tasks at hand.

One reason for the disparate findings in the literature may be the fact that social sensitivity might not apply to all adolescents but might be an individual disposition across development (Do et al., 2020b). For instance, individual differences in peer resistance explained variance in findings about peer presence increasing (e.g., Chein et al., 2011) or decreasing the number of risky choices (Kessler et al., 2017) depending on the task context used. Some reviews in recent years have pointed out that adolescents’ tendency to choose risky options is overly sensitive to diverse aspects of the task context (Defoe et al., 2019; Romer et al., 2017; Shulman et al., 2016). Apart from the social context, the sensitivity to risk probabilities has been shown to guide risk-taking tendencies during decision-making (Defoe et al., 2019). In classical experimental settings, participants may be able to deduce specific task outcomes, leading to unrealistic measures of risk-taking tendencies. To better approximate real-life decision-making, researchers increased uncertainty by obscuring outcome probabilities. Furthermore, researchers can create more dynamic version of traditional decision making tasks by manipulating the trial-by-trial probabilities of positive or negative outcomes. Using similar dynamic approaches, recent findings indicate that adolescents readily increase risky choices as opposed to situations under known risks (Defoe et al., 2019; Lorenz & Kray, 2019), also known as ambiguity tolerance. Such as heightened ambiguity tolerance in youth, peer presence effects interact with adolescent decision-making only in situations where the probabilities of outcomes are ambiguous instead of explicit (Lloyd & Döring, 2019; but see Smith et al., 2014). However, peer presence effects do not fully explain adolescent decision making, leading to an increase of risky choices in experience based tasks (e.g., Chein et al., 2011), decrease of risky choices in tasks with dynamically changing risk probabilities (Kessler et al., 2017) or having no effect on risky choice altogether (Reynolds et al., 2014).

Adding upon the sensitivity to the task context in adolescence, previous findings have highlighted that younger and older adolescents have divergent risk-taking tendencies. Consequently, inconclusive findings could also derive from the diversity in the age ranges and groups used to test peer presence effects. According to the suggested quadratic age trends in risk-taking (Shulman et al., 2016), adolescents showed greater tendencies for risky choices than adults in ambiguous risk situations. In contrast, adolescents showed similar risk-taking tendencies as children (for a review, see Defoe et al., 2019). Simultaneously, the boundaries of adolescence are shifting, a development that can be observed in science but also in legal and health policies. On the one hand, there is an ever earlier beginning of puberty and, on the other hand, neurodevelopmental findings push the upper threshold of the adolescent phase into the early 20s (see Ledford, 2018). To test developmental differences in peer presence effects, most studies have assessed peer presence effects form one narrow adolescent age group and have compared adolescent groups with adult groups at utmost. Yet, a few studies have provided further evidence that there is a high variability in peer presence effects between decision contexts even within the adolescent period. Effects of peer presence (aged 13–25 years, Somerville et al., 2019) and advice (aged 12–22 years, Braams et al., 2019) increased or decreased the number of risky choices dependent on the risk uncertainty in a given task context and the developmental stage throughout early to late adolescence.

One additional reason for inconclusive findings on peer presence effects might be the traditional calculation of dependent measures. Most studies rely on the mean number of risky choices that have been tightly associated with risk-taking tendencies. Even the names of many decision-making tasks refer to the term risk, like the Balloon Analog Risk Task (BART, Lejuez et al., 2002). Thereby, risk-taking is traditionally calculated by the mean number of risky choices for trials that resulted in positive outcomes (adjusted mean number of risky choices) to compensate for censoring, as trials end early in case of negative outcomes in the BART. But in contrast to decision-making under known risk where trials are independent probabilistic events, peers might influence how adolescents use information to update subsequent decisions, especially in uncertain and dynamically changing risk environments. Taking the inherent variability of the sample, developmental differences in behavioral adjustment to risk uncertainty throughout pre- to late adolescence were considered in this study. In order to model a realistic environment, a sequential decision-making task under risk uncertainty was used, the BART (Lejuez et al., 2002). The BART is a dynamic version of experience-based decision-making where participants are instructed to inflate virtual balloons. Each pump signifies a simultaneous increase in potential monetary outcomes, as well as a risk of bursting and the loss of all previous earnings.

Reinforcement learning analyses are particularly well suited to modeling such sequential decision-making (for the BART, see Wallsten et al., 2005) as they allow to distinguish between processes that have previously been discussed to contribute to behavioral adjustment, like exploration and learning. To model choice behavior, reinforcement learning models assume that participants estimate the value of each choice option and update these estimates according to experiences made. In the example of the BART, a parameter is calculated that indicates the participant’s belief in success when pumping a balloon, i.e., the individual belief that pumping a balloon may lead to monetary gains. Based on the belief in success, an updating rate is calculated that quantifies how fast participants adjust their beliefs in success to actual choice outcomes. Risk-taking tendency in this example is the tendency to pump above the perceived value of pumping or saving previous earnings. Moreover, the so-called inverse temperature estimate determines the extent to which the different values of choice options guide choice behavior. High inverse temperature values mean that the difference in outcomes of choice options is exaggerated and individuals stay with a specific choice pattern. In contrast, a low inverse temperature score can reflect exploration tendencies, i.e., the tendency to try out different options and to gather information when in uncertainty (Nussenbaum & Hartley, 2019). In sum, formal models allow for more specific hypotheses on behavioral adjustment that could help to uncover the effect peers have on risk-taking but also on adaptive behavior. In this sense, reinforcement learning analyses have successfully been applied to identify subgroups with addictive tendencies (Wallsten et al., 2005).

More specifically, constructs assessed through the parameters of formal models can be compared between different choice architectures, groups, or states (Nussenbaum & Hartley, 2019), like peer presence. One potential hypothesis would be that peer presence would provoke impulsive behavior and associated risk-taking by increasing the perceived potential for rewards during adolescence. This is the case because adolescents have been assumed to be specifically reward sensitive due to only gradual increases in cognitive control abilities but a rise in susceptibility to rewarding cues (Shulman et al., 2016). Unlike adults, adolescents showed greater activity in reward-related brain systems while engaging in a higher number of risky choices in a study using simulated driving under peer observation (Chein et al., 2011). Despite evidence on the neural level, findings on the behavioral level have shown evidence against the suggestion that adolescents are reward-sensitive in all situations (for a review, see Kray et al., 2018). When a peer observed choice behavior in the BART, adolescents were even more cautious and reduced the number of risky choices in trials after positive outcomes (Kessler et al., 2017). An alternative hypothesis could be that a high number of risky choices rather reflects exploration tendencies and learning from experiences instead of rash and impulsive choice behavior (Romer et al., 2017). Late adolescents (aged 18–21 years) explored choice options and used both, positive and negative feedback, to adjust behavior towards long-term goals when a peer observed choice behavior in a gambling task. When risks and outcomes could only be experienced, adolescents increased adaptive decision-making through exploration behavior and learning from its outcomes (Silva et al., 2016). Thereby, decreasing exploration tendencies with age across the lifespan is one of the most robust findings in developmental studies applying reinforcement learning models (Nussenbaum & Hartley, 2019).

Finally, an additional factor that has been shown to interact with exploration tendencies and peer susceptibility is gender. Female adolescents have been shown to be less sensation-seeking than male adolescents, and thus, less inclined to show exploratory behavior and associated risk-taking (Cross et al., 2011). Similarly, male adolescents have engaged in a higher number of risky choices than female adolescents in several decision-making tasks (de Boer et al., 2017; Cazzell et al., 2012; Lejuez et al., 2002; but not Lejuez et al., 2003) and self-described risk propensity measures (Byrnes et al., 1999). Given that peer presence can be a particularly arousing situation, male adolescents might also engage in more risk-taking than female adolescents in social situations. Some studies indeed have found risk-heightening effects of peer presence only for male adolescents (Defoe et al., 2020), or have found male adolescents to be at least more influenced by peer advice than female adolescents (Boer et al., 2017). However, other studies found no gender differences in peer presence effects on experimental risk-taking at all (Boer & Harakeh, 2017; Harakeh & Boer, 2018). In this sense, a qualitative review on gender differences in adolescent susceptibility to deviant peer pressure suggested male adolescents to be more influenced by peers than female adolescents in only 46% of all studies investigated (see McCoy et al., 2019). Together with exploration tendencies, developmental, and individual differences in social susceptibility, gender identity seems to add to the number of factors that exert a non-linear, dynamic influence on adolescent risk-taking in peer presence.

## Current Study

It has been assumed that peer presence increases risk-taking tendencies, specifically in adolescence, but findings of peer presence effects on experimental decision-making have been inconclusive. Inconsistencies in findings on peer presence effects could derive from the fact that the behavioral adjustment to positive and negative outcomes of risky decision-making, as well as individual differences in age, gender, and susceptibility to peer influences have seldom been considered. In this study, experience accumulation was assessed from adolescents of a large age-range while peer observation was present or absent during a decision-making task in which risk probabilities change dynamically. If peer presence increases risk-taking in adolescence, the number of risky choices, as well as risk-taking tendencies, should be higher when peer observation is present than when it is absent. As it has been assumed that this might be due to a higher prospect of rewards under peer presence, effects of peer observation should be maximal following positive outcomes and increase the belief in success. In contrast, peer observation could rather affect experience accumulation than risk-taking tendencies, either through faster learning and/or an increased exploration of positive and negative outcomes of risk. Moreover, it can be assumed that peer observation influences choice behavior to a greater extent in mid-adolescence (quadratic age trend), male adolescents and subjects with a low resistance to peer influence as opposed to pre- and late adolescents, female adolescents, and individuals with a high resistance to peer influences.

## Methods

### Participants

Overall, 193 participants were invited to be part of a larger longitudinal study that investigated the development of cognitive control and motivational functioning at two time points from early to late adolescence (age range = 9–19 years at T1). This study includes the cross-sectional data at T1. Participants were recruited via flyers and newspaper advertisements or were invited from the subject pool of the research unit in which the study was conducted and were paid a monetary compensation of 8 € per hour. Ethical approval for the project was given by a local ethics committee. Four participants had missing data in the risky decision-making task due to technical issues and five participants did not fill out the questionnaire used in this study. As the order of peer observation condition was counterbalanced, the participants with missing data were from different age groups, and the missing data accounted for below 5% of the whole dataset, complete case analysis was used by excluding participants with missing data from all analyses. Age was treated as a continuous variable in all analyses except for preliminary analyses in which age was binned into ten age groups from age 9 to 18. The mean age of the final sample (*N* = 184) was 14.09 years (min = 8.59, max = 18.97, *SD* = 2.95, 47% female^1^). The gender distribution was similar across the age groups, *χ*^2^(9, *N* = 184) = 10.92, *p* = .281. The final sample is further described in Table 1.

**Table 1:** Descriptive statistics, means, and standard deviations in choice behavior and resistance to peer influence of the final sample

		Gender	Age		Pumps		Bursts		RPI score	
Age group	*n*	% female	*M*	*SD*	*M*	*SD*	*M*	*SD*	*M*	*SD*
9	20	30	9.4	0.4	22.2	9.1	12.3	5.7	2.8	0.4
10	15	27	10.6	0.2	25.4	11.0	12.8	6.0	3.1	0.3
11	16	50	11.6	0.3	21.7	7.9	10.8	4.8	2.9	0.5
12	21	38	12.5	0.3	27.7	8.7	14.7	4.8	2.9	0.4
13	21	48	13.6	0.3	25.5	9.3	14.4	5.9	2.9	0.5
14	17	47	14.5	0.3	30.8	9.8	18.2	7.9	3.0	0.5
15	18	44	15.5	0.3	29.0	10.3	14.8	5.3	3.1	0.4
16	13	54	16.5	0.3	28.4	5.4	14.5	3.8	2.8	0.5
17	23	57	17.5	0.2	28.3	9.8	14.8	6.9	3.0	0.4
18	20	70	18.5	0.3	26.2	8.6	13.5	4.0	2.8	0.4

Age was binned into ten age groups for illustrative purposes only. RPI score = Score from the Resistance to Peer Influence Scale.

### Material

#### Balloon Analog Risk Task

**Fig. 1:**
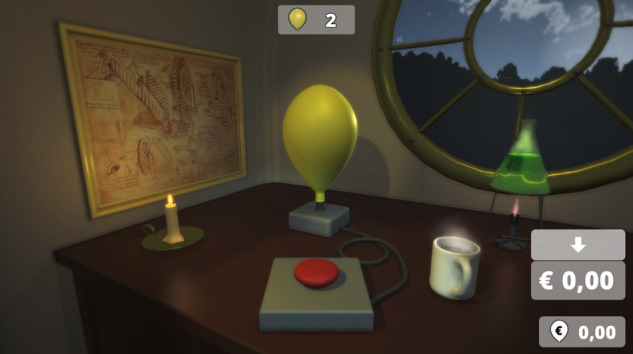
Illustration of the BART environment. Participants were instructed to gain as much money as possible by inflating balloons. Each balloon was treated as a trial and the number of balloons left was visible in the middle of the upper part of the screen. At any time, participants had to decide to either pump a balloon by pressing the button in the middle of the screen (space key) or to save the amount gained with previous pumps (down arrow key) and begin with a new balloon. The virtual account and amount of money earned with the previous balloon were visible on the upper right of the screen. Each pump increased the outcome of a balloon by 5 cents. However, participants were informed that the balloon could burst at a random inflation point and that all temporary gains would be lost if not saved to the virtual account. No further information, i.e., about burst probabilities, was given

The Balloon Analog Risk Task (BART, Lejuez et al., 2002) is a decision-making task under dynamic risk, as participants must weigh the potential monetary gain when pumping a balloon against the increasing risk of it to explode (probability of 1/128-n in the n-th trial). Each pump signified a gain of 5 cents and participants were instructed to collect as much money as possible. Money pumped into a balloon could be saved on a virtual account but was lost if the balloon exploded before doing so. Participants were told that the monetary gain for balloons increased with each pump but that balloons could explode at any point without referring to explicit probabilities. The structure of the original BART was not changed but the presentation of its balloon environment (see Fig. 1). Balloon explosions were presented in picture and sound and participants had insight in how many of their balloons (trials) were left, how much money was on their virtual account, and how much money they gained with the previous balloon. The BART was conducted on a computer using a 19-inch monitor and the computer keyboard. Balloons were inflated via a key press. Pressing the key activated an animation showing a red button that was connected to the balloon to be pressed which inflated the balloon. The participants performed the BART consecutively under two conditions: alone and under the observation of a fictitious peer (see section *Virtual Peer Observation*). The sequence in which participants conducted the task conditions was counter-balanced for each of ten age groups (age 9 to 18). Participants inflated 30 balloons and 3 practice trials in each of the two peer observation conditions. Overall, participants’ number of pumps was counted for 60 balloons that were treated as separate trials.

#### Virtual peer observation

**Fig. 2:**
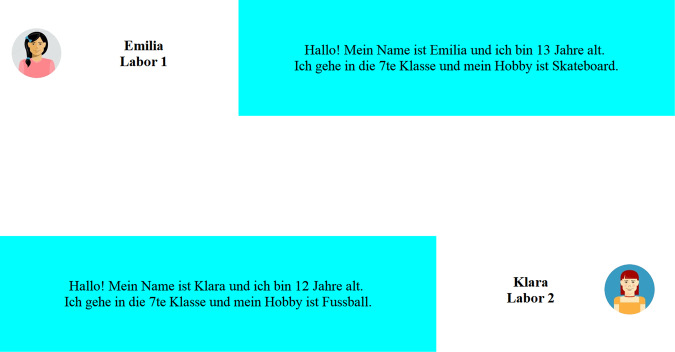
Illustration of the Chat environment as seen by a female participant. Participants were informed about a peer who would observe them via webcam during the conduction of the Balloon Analog Risk Task (BART). Before the peer observation condition block, they were introduced to the peer via a chat environment. Participants provided information about their name, age, grade, and hobbies in an otherwise preformulated chat message. Unbeknownst to the participants, the peers' answer that appeared after a short period was a randomly generated text message that matched the participants' information about gender and grade, as well as age by plus/minus one year

To assess whether adolescent risk-decisions are influenced by the presence of peers, a virtual same-age, same-sex peer was introduced via chatroom manipulation (see also Haddad et al., 2014; Smith et al., 2014). As such, adolescents conducted the BART once when peer observation was absent and once believing that a peer would observe them via a webcam. A program was used that broadcast the webcam recording and a screen mirror to the second laboratory (OBS Studio, 2015). Though its function was visible for the participant, the program was only started to increase the credibility of the peer scenario without actually broadcasting to another location or recording. To introduce the peer, participants were told that they will chat with a peer sitting in another laboratory before starting the task. In the virtual chatroom (see Fig. 2), participants provided information about their name, age, school year, and a hobby from which information a chat message was automatically formulated and supposedly sent to the peer. Unbeknownst to the participants, the answer that appeared after a short period was a randomly generated text message that matched the participants’ information about gender and grade, as well as age by plus/minus one year. Afterwards, participants were told that the BART is starting and their performance and the webcam recording would now be broadcasted to the other location. After the peer observation condition, participants were told and shown that the internet connection and the broadcast tool will be shut down and that all other tasks will be conducted without peer observation. The gender distribution was similar between groups of participants that conducted the BART either under peer observation first or second, *χ*^2^(1, *N* = 184) = 1.36, *p* = .244. Moreover, there was no difference in the distribution of participants that performed the BART under peer observation first or second across ten age groups, *χ*^2^(9, *N* = 184) = 5.96, *p* = 0.744.

#### Resistance to Peer Influence

A German version of the Resistance to Peer Influence Scale (RPI, Steinberg & Monahan, 2007) was administered to measure the extent to which participants describe themselves as being susceptible to peer influences. In this questionnaire, participants had to choose which of two statements best described their general predispositions (e.g., “Some people go along with their friends just to keep their friends happy.” BUT “ Other people refuse to go along with what their friends want to do, even though they know it will make their friends unhappy.”). After indicating one of the two options that described them best, they were asked whether this description is “really true for me” or “sort of true for me.” The RPI score is then calculated from participants’ choices in 10 pairs of statements. High RPI scores indicated a high resistance to peer influence. In this study, the RPI had a reliability index of 0.67 for the whole sample, suggesting the measure to be consistent across items and participants. The mean RPI score of 2.93 was comparable to previous studies that included the RPI measure in adolescent samples (e.g., Kessler et al., 2017). Additionally, the score differed neither between genders, *F*(1,176) = 0.38, *MSE* = 0.18, *p* = 0.537, ${\hat{\eta }}_{G}^{2}$ = 0.002, nor between groups that conducted the BART under peer observation first or second, *F*(1,176) = 0.02, *MSE* = 0.18, *p* = 0.875, ${\hat{\eta }}_{G}^{2}$ = 0.0001, and showed no age differences, *F*(1,176) = 0.002, *MSE* = 0.18, *p* = 0.967, ${\hat{\eta }}_{G}^{2}$ = 0.00001.

### Procedure

Data were collected as part of a comprehensive cross-sectional and longitudinal study on the interplay between motivational and cognitive control processes during adolescent development. At each of the two test points, participants took part in three sessions. In the first session, participants received a comprehensive test battery testing cognitive control functioning and decision-making, including the BART that is part of the present study. In the second and third session, participants furthermore conducted two tasks during which electrical brain activity was measured. Between the sessions, participants further completed various online self-report questionnaires via the online survey platform SoSci Survey (Leiner, 2019). These questionnaires collected information about, for example, demographic characteristics, or traits such as resistance to peer influence, and were filled out at home between the sessions. The instructions of these questionnaires requested the participants to ask the research team or their parents if problems occurred, but to complete the questionnaires preferably undisturbed. Please note that performance in the BART did not increase or decrease the monetary compensation of 8 Euro per hour. Yet, participants were told that depending on their performance in the BART and two other decision-making tasks (Stoplight, and Treasure Hunting Task) they would receive a gift, like pens, notepads or toys. For further information about the study procedure, see the *Supplementary Material*.

### Data Analysis

To assess the trial-by-trial adaptation in the BART, the raw number of pumps per trial was used. Additionally, a formal model on the *Number of Pumps* was applied to quantify expectations and behavioral adjustment based on a series of previous choices and outcomes that also reflects incremental processes, like exploration tendencies and learning in the BART (Wallsten et al., 2005).

#### Computational modeling

The computational model was fitted for the two peer observation conditions (Peer Observation absent/present) to investigate the effect of peer observation on the resulting constructs. To this end, the *hBayesDM* package, a toolbox that implements hierarchical Bayesian parameter estimation using STAN in the R environment (for further details, see Ahn et al., 2017), was used. Overall, 800 samples were used including 400 burn-in samples per chain to estimate the four parameters by converging them to their target distributions. To assure that the posterior distributions (the resulting parameters) were not dependent on the initial starting point, the parameter estimation ran on six independent chains. All $\hat{R}$ values for the parameters of interest were equal to or below 1.01, which suggests convergence of the parameters to their target distributions. The number of effective samples were above 100 for all parameters of interest and the trace plots indicated that the chains were well mixed. The exact equations for the following four constructs to be estimated are described in the Supplementary *Appendix*.

1. **Belief in Success (phi:** *ϕ***):** The *Belief in Success* reflects the belief that the balloon will not burst and results in positive outcomes. A high value indicates a prospect of rewards when taking the decision to pump.
2. **Learning (eta:** *η***):** The updating rate of the *Belief in Success* indicates the pace of *Learning* from previous outcomes. A high value indicates the tendency to adjust choices to previous outcomes.
3. **Risk-Taking (gamma:** *γ***):** *Risk-Taking* indicates the preference to pump a balloon despite the subjective utilities for pumping or not-pumping. A high value signifies the tendency to pump balloons irrespective of whether a positive or negative outcome is expected.
4. **Exploration Tendencies (tau:** *τ***):** The inverse temperature indicates to what degree previous experiences guide behavior. A low inverse temperature indicates ‘noisy’ behavior, a pattern that has been attributed to greater *Exploration Tendencies*, while a high value indicates the tendency to exploit choice options with maximal subjective utility.

#### (General) linear mixed models

As the *Number of Pumps* is a count variable, the logarithm link function with a Poisson distribution was applied using the *glmer* function of the *lme4* package (Bates et al., 2015) in *R* (Version 4.0.5, R Core Team, 2021). Within-subject factors and their interactions (the intercept, Peer Observation, as well as Trial Number, Previous Outcome, and their interaction with Peer Observation) were included as random effects, i.e., were allowed to vary across subjects. Analyses on the parameters of the computational model were calculated using the linear mixed model function (*lmer*) of the *lme4* package and included a random intercept per participant. P-values were estimated via the Satterthwaite approximations to degrees of freedom with the *lmerTest* package (Kuznetsova et al., 2017). Further details of the linear mixed models are described in the Supplementary *Appendix*. Developmental differences (Age, continous, age range = 8.59–18.97 years, mean age = 14.09 years) in the effect of peer observation conditions (Peer Observation, coded as present: 1; absent: -1) were tested on the *Number of Pumps*, *Belief in Success*, *Learning*, *Risk-Taking*, and *Exploration Tendencies*. A gender term (Gender, coded as male: 1; female: -1), as well as its higher and lower order interaction with Age and Peer Observation, were also included in the models. Individual differences in resistance to peer influence (RPI, mean scaled, range = 1.70–3.80, *M* = 2.93) and its interaction with Peer Observation were included in the predictions. The adjustment to positive and negative outcomes (Previous Outcome; coded as burst: 1; cash: -1), trials (Trial Number; coded 2 to 30 2; mean scaled for each peer observation condition), and interactions with Peer Observation and Age were included in the analysis on the *Number of Pumps*.

## Results

Mean values and standard deviations of the *Number of Pumps* for ten age groups can be found in Table 1. Adolescents pumped on average 26.50 times (*SD* = 9.34) per balloon with 14.12 balloons (*SD* = 5.84) that burst and an virtual outcome of 61.88 € (*SD* = 18.11) across overall 60 balloons. Thereby, the mean number of pumps (26.50) indicated rather risk-averse behavior as the number was significantly lower than the maximum earnings point (64 pumps, see *Figure 1* in the Supplementary *Appendix*). The estimates, standard deviations, significance values, as well as estimates for all fixed effects of the final model calculated on the *Number of Pumps* can be found in Table 2. As reported in Table 2, estimates explained little variance in the data, as measured by marginal variance (*R*^2^ = 0.08). However, the 95% confidence intervals were narrow, suggesting precise estimates. Thereby, a conditional *R*^2^ of 0.85 indicated that the subject variance was well captured by the random structure, suggesting that the application of random slopes and intercepts was appropriate. For the linear mixed models on parameters of the computational model, a complete summary of model estimates for all parameters can be found in Tables 3, 4, 5, and 6, of the *Supplementary Material*.

**Table 2:** Estimated model fixed effects, confidence intervals, significance values, and model fit of the Poisson regression on choices in the BART.

	Model linear age: Pumps		
Predictors	Estimates	Conf.Int(95%)	p value
(Intercept)	3.13	3.07–3.19	<**0.001**
Peer Observation [absent]	–0.02	−0.04 to 0.01	0.127
Age	0.11	0.05–0.17	**0.001**
Trial Number	0.02	0.01–0.04	**0.002**
Previous Outcome [cash]	0.09	0.08–0.10	<**0.001**
Gender [female]	–0.07	−0.13 to 0.01	**0.032**
RPI	0.04	−0.02 to 0.10	0.199
Peer Observation * Age	0.02	−0.01 to 0.04	0.171
Peer Observation * Trial Number	0.00	−0.01 to 0.02	0.706
Peer Observation * Previous Outcome	0.01	−0.00 to 0.02	0.106
Peer Observation * Gender	–0.01	−0.03 to 0.01	0.211
Age * Trial Number	0.01	−0.01 to 0.02	0.364
Age * Previous Outcome	–0.01	−0.02 to 0.01	0.416
Age * Gender	0.00	−0.07 to 0.06	0.876
Peer Observation * RPI	0.02	0.00–0.04	**0.027**
Peer Observation * Age * Trial Number	–0.02	−0.03 to 0.00	**0.027**
Peer observation * Age * Previous Outcome	–0.01	−0.02 to 0.01	0.373
Peer Observation * Age * Gender	0.01	−0.01 to 0.03	0.456
ICC	0.83		
N ID.Age	184		
Observations	10672		
Marginal R2 / Conditional R2	0.079 / 0.845		

Significant effects are highlighted in bold. *p* values were estimated via the Satterthwaite approximations to degrees of freedom.

### The Effect of Peer Observation on Decision-Making

**Fig. 3:**
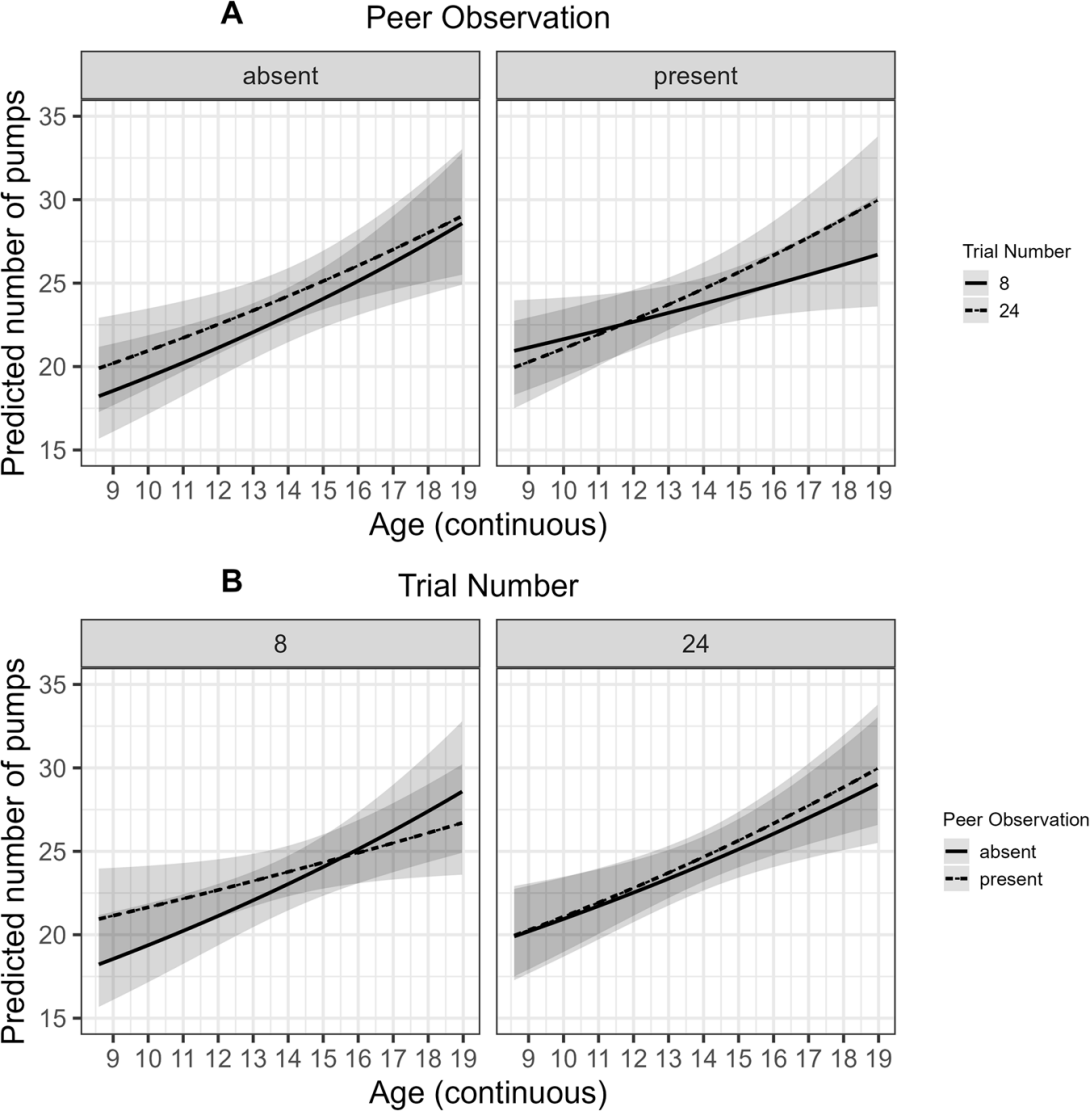
Predicted results of general linear mixed effects regression (glmer) on the number of pumps for the peer observation conditions across age and trials. **A** Age trends (age range = 9–19 years, mean age = 14 years) in the predicted number of pumps as a function of Trial Number (range 2–30; normalized for each condition) for the two peer observation conditions (absent/present; −1/1). **B** Age trends (range = 9–19 years, *M* = 14 years) in the predicted number of pumps as a function of the peer observation conditions (absent/present; −1/1) for early and late trials (Trial Number; range 2–30; normalized for each condition). The predicted values and error bands (standard errors) are from the final model with continuous age in its original scale. The effects plots are averaged across Previous Outcome, RPI, and Gender

The trial-by-trial regression revealed no main effect of Peer Observation on the *Number of Pumps* but there was a higher-order interaction between Peer Observation, Age, and Trial Number (see Table 2 and Fig. 3). There was also a main effect of Previous Outcome suggesting that adolescents reduced the *Number of Pumps* following trials in which the balloon exploded. There was no interaction between Previous Outcome and Peer Observation or Age (see Table 2). The *Belief in Success* showed no significant effects (all *p*’s ≥ 0.070). *Risk-taking* (*γ*) was higher when Peer Observation was absent than when it was present, *b* = 0.02, *SE* = 0.01, *p* = 0.030. That is, Peer Observation decreased the tendency to engage in pumps irrespective of the expected value of pumping a balloon further. There were no other main or interaction effects observed for *Risk-Taking* (all *p*’s ≥ 0.145). The *Learning* parameter showed no significant effects (all *p*’s ≥ 0.054). Overall, Peer Observation decreased instead of increasing *Risk-Taking* and the findings suggest developmental differences in the effect of Peer Observation on the sampling of trials (see section *Developmental Differences in the Effect of Peer Observation*).

### Developmental Differences in the Effect of Peer Observation

There was a higher-order interaction between Peer Observation condition, Age, and Trial Number but no main effect of Peer Observation and no lower-order interactions between Peer Observation, Age, or Trial Number on the *Number of Pumps* (see Table 2). Visual inspection of the effects plots revealed that there was an interaction between Age and Trial Number but only when Peer Observation was present and an interaction between Age and Peer Observation only at early trials (see Fig. 3). Under peer observation, the increase in the *Number of Pumps* with the Trial Number was steeper in older adolescents than younger adolescents (see Fig. 3A). At early trials, younger but not older adolescents engaged in a higher *Number of Pumps* when peer observation was present than when it was absent (see Fig. 3B). On the level of the mean, the *Number of Pumps* increased with Age and Trial Number (see Table 2). Developmental differences in the effect of Peer Observation on adjustment to task experiences were accompanied by developmental differences in *Exploration Tendencies* but these were modulated by Gender (see Section *Gender and Individual Differences in the Effect of Peer Observation*).

### Gender and Individual Differences in the Effect of Peer Observation

**Fig. 4:**
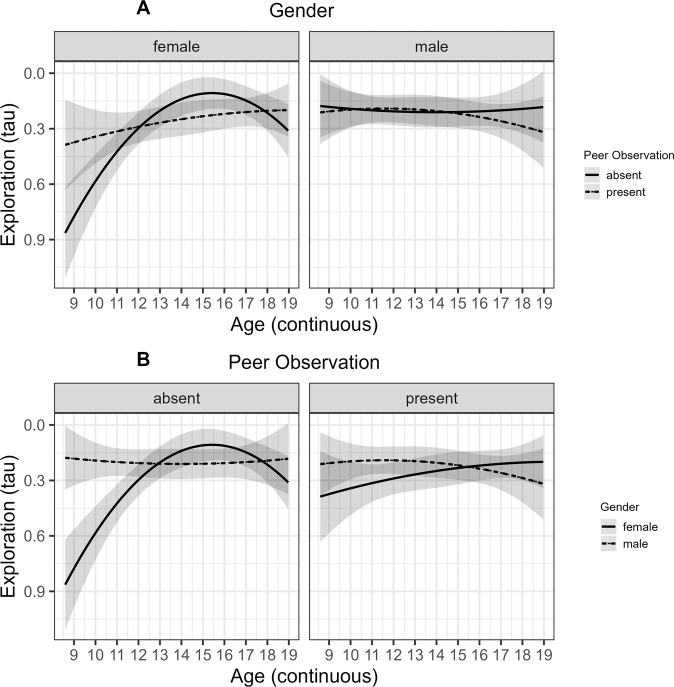
Predicted results of linear mixed effects regression (lmer) on exploration tendencies for the peer observation conditions and genders across age. **A** Moderation of gender (female/male; −1/1) on age trends (age range = 9–19 years, mean age = 14 years) in the effect of peer observation conditions (absent/present; −1/1) on exploration tendencies during the Balloon Analog Risk Task (BART). **B** Moderation of peer observation condition (absent/present; −1/1) on differences in age trends (age range = 9–19 years, mean age = 14 years) between the genders (female/male; −1/1) in exploration tendencies during the Balloon Analog Risk Task (BART). The predicted values and error bands (standard errors) are from the final model with continuous age in its original scale. Predicted values are averaged across RPI scores and the y-axis was inverted to reflect exploration tendencies

**Fig. 5:**
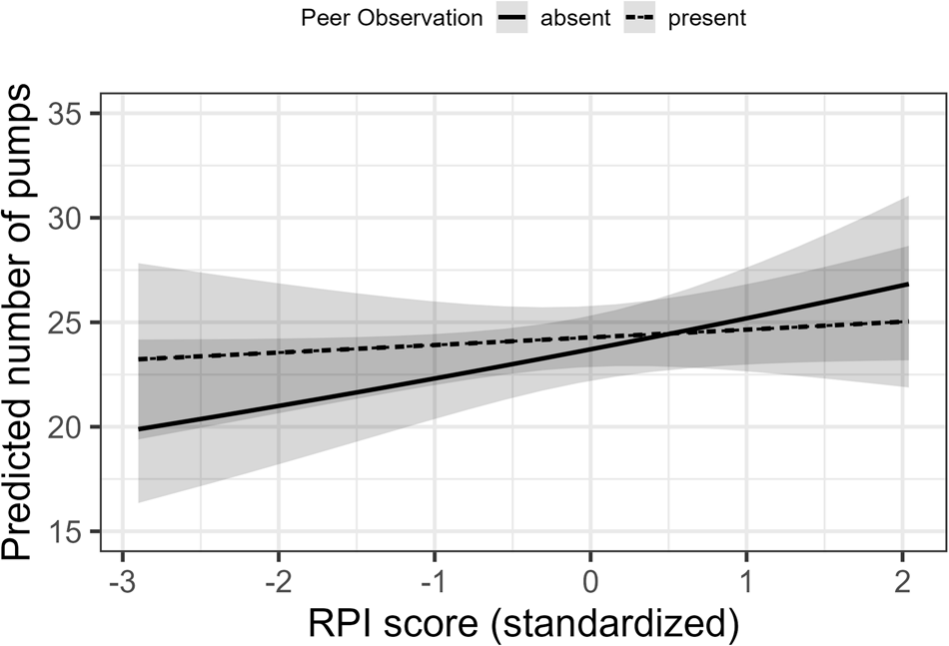
The effect of peer observation condition (absent/present; −1/1) in the predicted number of pumps of the Balloon Analog Risk Task (BART) as a function of resistance to peer influence (RPI) scores (standardized). The predicted values and error bands (standard errors) are averaged across Age, Trial Number, Previous Outcome, and Gender

For the parameter reflecting *Exploration Tendencies* (*τ*), there was a higher-order interaction between the quadratic age term, Peer Observation, and Gender, *b* = 0.69, *SE* = 0.26, *p* = 0.008. Female adolescents showed a peak in *Exploration Tendencies* during mid-adolescence but only when peer observation was absent (see Fig. 4). A lower-order interaction between Age and Condition, *b* = −0.55, *SE* = 0.26, *p* = 0.037, was accounted for by adolescents’ *Exploration Tendencies* being higher when Peer Observation was present than when it was absent at younger ages but differences between the peer observation conditions decreased until mid-adolescence. The higher-order effects plot revealed that this Age by Condition interaction was only given for female adolescents (see Fig. 4A). A lower-order Age by Gender interaction, *b* = −1.04, *SE* = 0.34, *p* = 0.002, indicated that young female adolescents had lower *Exploration Tendencies* than young male adolescents but gender differences decreased with age. The higher-order interaction suggested that an Age by Gender interaction was only observable when Peer Observation was absent (see Fig. 4B). This resulted in a main effect of quadratic age on *Exploration Tendencies*, *b* = 0.72, *SE* = 0.33, *p* = 0.029. No other main or interaction effects of Gender or RPI scores on parameters of the computational model could be observed. With regard to the trial-by-trial regression, male adolescents engaged in a higher *Number of Pumps* on the level of the mean than female adolescents but there were no interactions between Gender and Peer Observation or Age (see Table 2). Finally, there was a significant interaction effect between Peer Observation and RPI scores on the *Number of Pumps* (see Table 2) but no effects of RPI scores on the parameters of the computational model. Adolescents with low RPI scores showed a higher *Number of Pumps* in the peer observation present than absent condition (see Fig. 5).

## Discussion

It has been discussed whether peers increase risk-taking tendencies by increasing the perceived potential for rewarding outcomes or whether other processes, like exploration tendencies and learning, are involved in peer presence effects on adolescent decision-making. In this study, the BART was applied to investigate the dynamic nature of adolescent risk-taking under peer observation. Unlike traditional approaches, adolescent behavior was modeled on a trial-by-trial basis accounting for the influence of peer observation on different cognitive processes during decision-making, namely the belief in success, learning, risk-taking and exploration tendencies. Furthermore, individual differences in age (linear and quadratic), gender, and resistance to peer influences were considered as potential moderators. In contrast to the assumption that peer presence heightens risk-taking tendencies in adolescence, peer observation decreased risk-taking but increased exploration tendencies. Adolescents increased the number of risky choices with age but peer observation effects were most prominent in female preadolescents. Though male adolescents showed a higher number of risky choices and greater exploration tendencies on the level of the mean, it was the exploration tendency of women that showed a mid-adolescent peak and increased as an effect of peer observation. As hypothesized, individuals with low self-described resistance to peer influence engaged in a higher number of risky choices under peer observation. In sum, the findings emphasize to consider cognitive processes outside of risk-taking tendencies and the importance to study person-environment interactions when investigating peer effects in adolescence.

### How does peer observation influence choice behavior in the BART?

In this study, risk-taking tendencies were significantly lower in the peer observation present than in the peer observation absent condition. This finding suggested that peer observation caused more cautious behavior in the BART. More specifically, the definition of risk-taking tendencies in the computational model implies that adolescents reduced the number of pumps irrespective of whether this behavior would increase the expected value or not. This finding stands in contrast to developmental models that suggest peers increase risk-taking in adolescence (Shulman et al., 2016), such as during simulated driving (Chein et al., 2011). However, in line with the present findings, adolescents reduced their pumps under peer observation in the BART (Kessler et al., 2017). The Bayesian modeling approach assumes that decreases in the number of pumps might be generally due to decreases in risk-taking tendencies under peer observation. Accordingly, adolescents, like adults, have shown a number of pumps below the maximum-earnings point, suggesting rather cautious behavior in the BART (for a review, see Lauriola et al., 2014). In sum, the findings are in line with investigations that show that peer presence effects do not contribute to a straightforward increase in risk-taking in adolescence. One mediating factor of peer susceptibility in adolescence has been assumed to be the fact that peer presence increases the value of potential rewards (Shulman et al., 2016). However, the findings indicate that all adolescents adjusted their choice behavior to previous balloon explosions by reducing their number of pumps in the following trials, irrespective of peer observation. This is in line with previous studies that showed no effect of previous positive trial outcomes on adolescents’ beliefs about whether pumping a balloon will be successful, or not (Élteto et al., 2019). Under peer observation, male adolescents were even more cautious following successful trials in the BART (Kessler et al., 2017).

That is, peers might indeed increase the motivational value of a situation but this must not lead to negative outcomes in all situations during adolescence (Do et al., 2020b). As such, increases in risky choices can reflect adaptive behavior, as a higher number of risky choices can maximize potential gains in decision-making tasks. Moreover, peer presence might also motivate incremental processes, like learning and exploration. Learning and exploration might sometimes be causal to increases in the number of risky choices but also to an increase in experience and, thus, a better assessment of risk situations on the long run (Romer et al., 2017). By considering the trial-by-trial choice behavior in the BART, this study demonstrated that peer observation increased the exploration of possible outcomes and changed behavior as a function of collected information. Overall, differences in exploration and experience accumulation between the peer observation present and absent conditions were subject to individual differences in age, gender, and peer resistance that will be discussed in the following sections.

### Developmental Differences in the Effect of Peer Observation

The third hypothesis was that if risk-taking and peer presence effects are indeed adolescent-specific, risk-taking tendencies and the influence of peer observation thereupon should increase from pre- to mid-adolescence but decrease in late adolescence. However, age comparisons across the span of adolescence have remained scarce (but see Braams et al., 2019; Somerville et al., 2019). To directly test a mid-adolescent peak in peer presence effects, both the linear and quadratic age trends in a wide age range (aged 9–19 years) were included in the analyses. The results showed that the number of pumps per trial increased linearly from pre to late adolescence showing no peak in mid-adolescence (no significant quadratic trend). This finding corresponds with previous studies that have found the number of risky choices in the BART to be either age-insensitive during adolescence (Élteto et al., 2019; Lejuez et al., 2002; Lejuez et al., 2003) or to increase until late adolescence (e.g., Braams et al., 2019). The linear developmental trend of risky choices in the BART has been attributed to developmental changes in the linear accumulation of experiences, rather than to risk-taking tendencies in adolescence (Élteto et al., 2019). To disentangle the two accounts, the effect of peer observation on the adjustment to previous experiences was modeled in this study.

Taking into account peer observation on a trial-by-trial basis in the computational model, it was surprising to find age and peer observation not affecting the learning rate of the adolescent participants. One explanation might be that the dynamic risk levels of the BART might increase the difficulty to track choices and to learn from their positive and negative consequences, independent from cognitive maturity and the social context. As such, late adolescents increased their learning rate in a gambling task that provides more stable outcome probabilities than the BART (Iowa Gambling Task, IWT, Silva et al., 2016). Beyond and above differences in task characteristics, Silva et al., 2016 tested older ages (aged 18–21 years) than in this study. As a steeper increase in the number of pumps with trials in older than younger adolescents was found when peer observation was present, it could be assumed that influences of peer presence on learning rates might increase until early adulthood. In contrast, peers might enhance experience accumulation via more indirect routes in uncertain situations. Adolescents might be more inclined to overcome habitual responses to social cues (e.g., conformative behavior) to explore unknown information (e.g., by engaging in risk-taking, Do et al., 2020b). It has to be noted that this assumption does not imply that exploration happens in the absence of cognitive control but increases the opportunity to adapt to and learn from new situations during adolescence (Do et al., 2020b; Romer et al., 2017).

According to the view that peer presence could promote exploration tendencies, the findings revealed that exploration tendencies, unlike learning rates, increased as a function of peer observation and age in the BART. These findings are also in line with the study of (Silva et al., 2016) that showed peer observation to increase exploration tendencies in the IGT and other studies that suggest adolescents to be generally more inclined to explore uncertain environments than other age groups (for an overview, see Do et al., 2020b). Moreover, unlike previous assumptions in the literature that exploration is an adolescent-specific effect, pre-adolescents in this sample indicated an increased openness to explore choice options under peer observation. The one-trial-back analysis complemented the findings on exploration tendencies, as it also revealed an effect of peer observation on pre-adolescents who increased the number of risky choices but only in early trials. In sum, the present findings suggest that when there is little task and life experience, peer observation encouraged pre-adolescents to try out behaviors if in uncertainty about possible outcomes. Furthermore, the findings indicate that the differences in exploration and experience accumulation with and without the presence of peers were subject to individual differences in age, gender, and peer resistance that will be discussed in the following sections.

### The Role of Gender and Individual Differences in Peer Resistance

In this study, previous findings were replicated indicating that male participants engaged in a higher number of pumps than female participants (Cazzell et al., 2012; Lejuez et al., 2002) in an adolescent sample (but see Lejuez et al., 2002). Using reinforcement learning models, the findings of this study moreover showed subtle gender and developmental differences in the peer effect on adolescent risk-related decision-making. First, not male but female developmental trajectories accounted for a mid-adolescent peak in exploration and associated risky choices in the peer observation absent condition. Compared to male pre-adolescents, female pre-adolescents stuck with their initial number of pumps, which was previously referred to as exploitative behavior, and adjusted to male levels of exploration only with age. Secondly, on average male adolescents engaged in more pumps per trial but they were not susceptible to peer observation. In contrast, under peer observation female pre-adolescents reached the level of exploratory behavior of male adolescents of the same age. According to a lower number of risky choices in the BART, it has been assumed that female adolescents show less sensation seeking and exploration behavior than male adolescents (Cross et al., 2011). However, it might be that if the situation is perceived as specifically rewarding, young women are nonetheless inclined to explore risk behaviors (Romer et al., 2017).

There are divergent hypotheses about gender differences in susceptibility to peer pressure, either suggesting female adolescents to be more or less resistant to peer influences than male adolescents. The suggestion that female adolescents have a higher social sensitivity than male adolescents could explain the elevated exploration tendencies of female pre-adolescents under peer observation found in this study. Moreover, the female social sensitivity is rather seen as a competence instead of deviance but most studies on gender differences in the influence of peers are on deviant behavior (for a review, see McCoy et al., 2019). In this study, heightened exploration of choice outcomes might be rather a positive influence of peer observation that might explain why female participants were inclined to increase the exploration of pump outcomes and ultimately got closer to the task’s maximum-earnings point under peer observation. Yet, male adolescents on average engaged in a higher number of pumps and there is evidence that peer presence is more likely to affect male than female adolescents’ choices (Defoe et al., 2020). Variable findings on gender differences in risky behaviors could be explained by gender differences being highly dependent on age and domain. As such, the present findings contribute to a general decrease of gender differences with increasing age. Moreover, the engagement in specific risk-taking behaviors, like risky driving, is known to be dominated by men in statistics and studies about self-described risk behaviors (for a meta-analysis, see Byrnes et al., 1999). Therefore, it can be reasonably assumed that peer presence might accelerate the risk proneness of male adolescents in traffic situations and simulated driving (Defoe et al. 2020). Male adolescents were also less affected by the anticipation of social punishment but more responsive to the reception of monetary gains than female adolescents in incentive delay tasks (Greimel et al., 2018). All things considered, male adolescents might be more inclined to find ways to increase monetary gains irrespective of peer observation, while female adolescents show similar reactions only under peer presence or with life experience.

One other explanation for convergent findings on peer presence effects is that the adolescent who has a low peer resistance may be a more influential factor than age or gender differences (McCoy et al., 2019). The findings of this study revealed that adolescents who described a low resistance to peer influences engaged in more risky choices when peer observation was present than when it was absent. This is in line with previous studies that showed peer resistance to be associated with a higher number of risky choices during simulated driving tasks (Chein et al., 2011). Recent reviews of social susceptibility in youth and its neural underpinnings suggest that individual susceptibility to peer influence most likely interacts with the decision situation and type of peer influence (Do et al., 2020b). Thus, a heightened susceptibility to peer influence in youth could promote both maladaptive and adaptive behavior depending on the situation at hand. A higher number of pumps signifies greater monetary outcomes suggesting a positive outcome of low peer resistance under peer observation in the BART. But there were no differences in cognitive processing between individuals with low or high peer resistance in this study that could underline these assumptions, e.g., by showing better learning, greater exploration or risk-taking tendencies under peer observation.

### Strengths, Limitations, and Future Outlook

A main advantage of this study was that it took into account multiple factors that affect risk taking behavior in adolescence such as age, gender, and peer resistance. The findings showed increases in exploration with age and peer observation but decreases in risk-taking tendencies. As such, the rise in real-life risk-taking during adolescence might reflect a drive to explore new behaviors rather than to indulge in risk-taking. This is in line with ongoing attempts to reflect the rise in risk-taking as a normal process that ensures growth in experiences and wisdom during the adolescent period (Romer et al., 2017). In similar attempts to overcome the negative stereotypes of adolescent risk-taking, the flexibility in decision-making should be emphasized, suggesting that adolescent choice behavior (Defoe et al., 2019; Romer et al., 2017) and social influences (Do et al., 2020b) might differ depending on risk contexts. However, this points to the importance to consider differences in motivational investment when studying peer influences on risk-taking. Though the BART environment used in this study was adapted to be emotionally enriched and visually appealing (see Supplementary Appendix Fig. 1), participants’ performance was not associated with the final compensation which should be taken into consideration when interpreting the findings.

Specifically, pre- to late adolescents’ susceptibility to peer observation during risk decision-making was investigated in this study, while most previous studies compared, if at all, only few age groups and did not include younger samples (Defoe et al., 2019). Though developmental differences were most pronounced for exploration tendencies, an alternative explanation for the developmental differences in the effect of peer observation on the number of pumps might be that peer presence distracted younger adolescents influencing them to act more impulsively in the beginning, while adjusting to peer presence with time. This might be justifiable as younger adolescents have a lower level of cognitive development and, therefore, be more easily distracted by peer presence than older adolescents. The present study design did not allow to confirm the hypothesis of peers as conduits of active exploration as opposed to a distraction. One possibility to do so would be to also measure the pump rate, i.e., how much time adolescents spend evaluating the pros and cons of engaging in another pump in the BART. Overall, the present findings suggested individual differences in age, gender, and peer resistance, emphasizing that not all adolescents in all situations are susceptible to peer influences. This emphasizes the importance to consider individual and contextual factors when investigating adolescent risky decision-making. For example, to test a potential domain-specificity of gender differences in and peer influences on adolescent decision-making.

Finally, peer observation increased the openness to explore outcomes when pumping balloons in a task in which pump levels are mostly beyond the maximum earnings point (for a review, see Lauriola et al., 2014). As such, exploration of outcomes and an increase in the number of pumps can here be interpreted as a positive effect. Even though much effort was put into disentangling the different processes implied in choice behavior in the BART, the findings can inform the adaptiveness of peer influences to a limited extent. For example, the current study design does not allow us to make conclusions about whether this was a controlled behavior (see Do et al., 2020b). That is, a tendency for random answers would have similarly resulted in a higher exploration tendency as a higher rate of controlled sampling of the task environment under peer observation (Somerville et al., 2017). To differentiate between random and controlled sampling, peer reactions to effortful and effortless choice strategies could be alternated in future investigations.

## Conclusion

Despite inconclusive findings in experimental decision-making, the consensus in previous literature has been that peer presence increases risk-taking during adolescence. In this study, there is evidence that under peer observation, adolescents showed an increase in cautious behavior and exploration tendencies in a dynamic decision-making task. Moreover, individual differences in age, gender, and peer resistance modulated the effect of peer observation on risk-taking. Specifically, pre-adolescent women as well as participants with lower peer resistance were most susceptible to peer observation. Crucially, instead of increasing risk-taking tendencies, the presence of peer observation allowed participants to try out novel behaviors and experience both downsides and upsides of their risk-taking. The present findings underline how studies on experimental decision-making together with considering individual differences might help to further uncover constellations of persons and situations that could be dangerous but also growth-enhancing for adolescent development.

## Supplementary information

Supplementary Information

Appendix Revision

## Footnotes

1 Please note that gender was assessed as a binary variable. It was asked about a person’s psychological sense of their gender, i.e., about their gender identity.

2 Note: Trial 1 was excluded as there were no previous responses at the beginning of the two peer observation condition blocks.
